# Homomorphic Asymmetric Encryption Applied to the Analysis of IoT Communications

**DOI:** 10.3390/s22208022

**Published:** 2022-10-20

**Authors:** Juan Luis López Delgado, José Antonio Álvarez Bermejo, Juan Antonio López Ramos

**Affiliations:** 1 Department of Informatics, University of Almeria, 04120 Almeria, Spain; 2 Department of Mathematics, University of Almeria, 04120 Almeria, Spain

**Keywords:** asymmetric encryption, homomorphic encryption, IoT

## Abstract

In this paper, we describe the use of homomorphic encryption techniques in order to not only ensure the data are transmitted in a confidential way, but also to use the encrypted data to provide the manager with statistics that allow them to detect the incorrect functioning of a sensor node or a group of sensors due to either malicious data injection, data transmission, or simply sensor damage (miscalibration, faulty sensor functioning). Obtaining these statistical values does not need decryption, so the process is sped up and can be developed in real time. Operating the data in this way ensures privacy and removes the need to maintain a shared key infrastructure between the sensor nodes and the manager nodes that are part of the blockchain infrastructure. In this work, we focus on operations with the sensor nodes that provide data that will be, later, treated as part of the business logic in the agribusiness sector (for example), hence the importance of having fast checking mechanisms in terms of data quality. The results obtained on conventional configurations of sensor nodes encourage the use of this technique in the aforementioned infrastructure.

## 1. Introduction

Wireless sensor networks constitute an integral part of most Internet of Things (IoT) devices, and their presence nowadays in our lives is growing exponentially [1]. The application areas of this emerging technology go from the development of autonomous cars to e-health, defense, or smart cities [2]. The transmitted data through the wireless sensor network is transformed into information, which is used to make decisions. Therefore, in most cases, a key point is to ensure the security of communications, and this leads to focusing on security requirements [3], which include confidentiality, integrity of the data transmitted, authentication of the source, and non-repudiation. Achieving these objectives is of crucial importance [4], especially in an environment where no human interaction is present, as is proposed in the definition of the IoT [5] as an interrelated system of devices with the ability to transfer data over networks. In this way, providing security for IoT communications presents different challenges The first one is to to find a way to maintain security with a technology requiring very limited computing resources, data storage, and even power consumption. The use of lightweight cryptographic algorithms helps to achieve the security requirements as well as energy savings. A second point to consider is data processing. It could be that our IoT application interconnects a large number of IoT devices which generate a huge quantity of data that aim to make decisions in real time; thus, it is necessary that the architecture uses scales with a wide range of sensors and, at the same time, provides interoperability since the network could be heterogeneous, where some devices simply transmit data, whereas there exist others that could perform computations that help in decision making.

In [6], the authors introduce a solution based on blockchain technology that takes into account all the preceding requirements. The use of blockchain in IoT networks is not new (see, for instance, [7,8,9]). The proposal in [6] makes use of smart contracts and a distribution of sensors by groups, named “device farms”, that are composed by a group of sensor nodes that transmit the data and a detached node, called the bridge node, which acts as a means of connection between the sensor nodes and the nodes in charge of adding the information to the blockchain. Bridge nodes essentially pack the transmitted data to these other nodes. The data are protected by using a shared key among the nodes that add the information to the blockchain and the device farms.

Although the use of a private blockchain makes it difficult to forge a sensor node and thus the falsification of data, it is very important as well to detect possible damages or incorrect functioning of sensor nodes. This can be achieved automatically by using statistical information obtained from the transmitted data. The aim of the paper is to show how this statistical information could be computed by the bridge nodes without the necessity of decrypting the received messages, maintaining the confidentiality, and without increasing their workload significantly. To accomplish this, we use homomorphic encryption, which is a type of encryption that allows us to perform some mathematical operations on encrypted data, resulting in some other mathematical operations on the corresponding plaintext. This technique has previously been used in the IoT setting with different purposes, as in [9,10]. Here, we use a pubic key cryptosystem, more precisely, the Paillier cryptosystem [11], and its homomorphic property in such a way that only the node or server in charge of analyzing the transmitted data is able to access the information that allows it to analyze them. Every bridge node acts as a means for the sensor nodes to communicate with these main nodes or servers that can compute the information needed to perform a statistical analysis without decrypting the transmitted data, maintaining confidentiality throughout the whole communication process and using simple operations derived from formulas induced by the aforemetioned homomorphic property of this cryptosystem in such a way that the statistical analysis can be carried out during the communication.

## 2. Materials and Methods

### 2.1. Architecture

**Figure 1:**
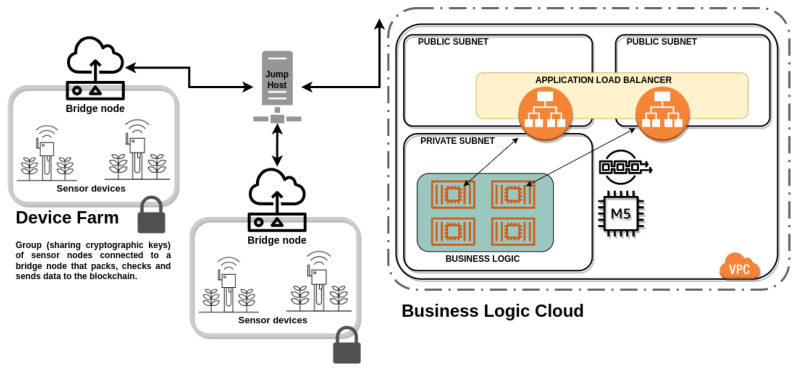
Computing infrastructure.

As previously noted, our aim is to preserve the confidentiality of communications between nodes and, following the ideas of [6], we will consider two types of nodes. On one hand, we have nodes that capture and transmit data and, on the other hand, the nodes that analyze them. This corresponds to the architecture of [6], shown in Figure 1.

A second option is that the so-called bridge nodes that simply package the received data and, from them, perform the corresponding computations that allows the server to analyze the transmitted data.

In the first case, every sensor node in a device farm should share a public key with its corresponding bridge node that allows it to authenticate the communications. The bridge node should share a unique public key with every sensor node in order to encrypt the collected data, and thus, only the bridge node will be able to retrieve the transmitted data. However, the bridge node should encrypt the collected data with the corresponding statistics that allow it to analyze them and send everything to the central server, but the bridge nodes, as it is pointed out in [6], have bigger capabilities than a simple sensor node.

In the other case, the central server is the only one authorized to retrieve the transmitted data, so these should be encrypted using its public key. Therefore, every sensor node should receive this public key, which can be achieved by means of any secure multicast protocol (cf. [12,13] and its references or, more recently, [14]). Then, the bridge nodes will act as virtual sensors by computing some needed data in order to perform an analysis.

We need to reduce the bridge nodes’ workload since, otherwise, this could slow down the communications: assume that every bridge node should collect the information sent by rest of sensor nodes in its device farm; then, the information should be encrypted with the bridge node’s public key or a session key in this group of some symmetric cryptosystem, and this bridge node should decrypt it; the statistical data are computed and encrypted again with a shared key with the central server, and finally, this key is sent jointly with the received data in its device farm. Thus, it is clear that an alternative would be desirable, which could be offered by homomorphic encryption.

### 2.2. Homomorphic Encryption

Homomorphic encryption is a form of encryption with an additional evaluation capability for computing over encrypted data without access to the secret key and the result of this computation is encrypted and can be reached by using the same key that would be used to decrypt the data involved in the computation. From the algebraic point of view, the encryption and decryption functions might be considered as applications that preserve the operations defined in the plaintext and ciphertext sets. This is not a new problem that arises by new technologies, but this kind of scheme was already proposed in [15].

**Figure 2:**
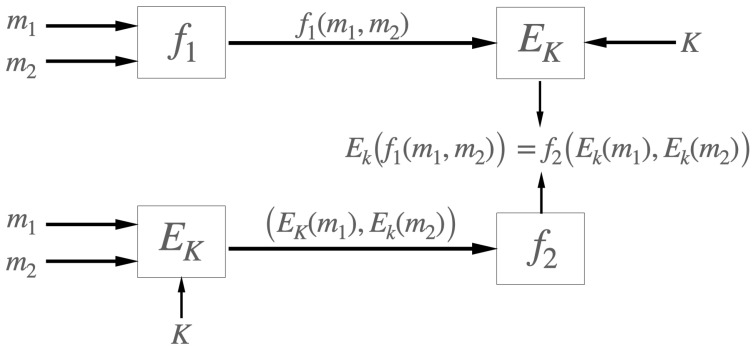
The encryption function $E_{k}:G_{1}\times K\to G_{2}$ takes as input a pair of values (g,k), where *g* is an element in a group $G_{1}$ and *k* an element in the space of keys and outputs an element of the group G2. The functions $f_{1}:G_{1}\times G_{1}\to G_{1}$ and $f_{2}:G_{2}\times G_{2}\to G_{2}$ define the two internal laws in the groups, $G_{1}$ and $G_{2}$, respectively.

The preceding is illustrated in Figure 2.

An immediate example of such a cryptosystem providing the homomorphic property is RSA. Thus, given a pair (n,e) defining the corresponding private key, it is clear that given $m_{1}$ and $m_{2}$, two messages are to be encrypted:

$$E\left(m_{1}\right)\cdot E\left(m_{2}\right)=m_{1}^{e}\cdot m_{2}^{e}\;\left(\mathrm{mod}n\right)={\left(m_{1}\cdot m_{2}\right)}^{e}\;\left(\mathrm{mod}n\right)=E\left(m_{1}\cdot m_{2}\right).$$

However, this property does not allow us any application to our purposes, given that most statistical measures, such as arithmetic mean, involve the addition of values.

#### 2.2.1. ElGamal-Type Cryptosystems

Let *G* a cyclic group and g∈G is a generator. Let us denote by ∗ the internal law in *G*. Let us consider $g^{a}$ as the public key for some positive integer *a*, which constitutes the private key. Then, the encryption of m∈G is given by the pair $\left(g^{k},m*{\left(g^{a}\right)}^{k}\right)$ for some random positive integer *k*. Here, $g^{n}$ means the operation *g* with itself *n* times.

Thus, we have that:

$$E\left(m_{1}\right)*E\left(m_{2}\right)=(g^{k_{1}},m_{1}*{\left(g^{a}\right)}^{k_{1}})*(g^{k_{2}},m_{2}*{\left(g^{a}\right)}^{k_{2}})=$$

$$(g^{k_{1}+k_{2}},\left(m_{1}*m_{2}\right)*{\left(g^{a}\right)}^{k_{1}+k_{2}})=E\left(m_{1}*m_{2}\right)$$

In the particular case of the group of points of an elliptic curve whose internal operation is an addition, which is a very convenient setting to be used in devices with light capabilities, the reader might think that this property would lead to an interesting formula to be applied with statistical purposes: $E\left(m_{1}\right)+E\left(m_{2}\right)=E\left(m_{1}+m_{2}\right)$. However we have to take into account that messages are, in fact, points of the elliptic curve ([16,17]), and the standard processes to assign a message to a point of the elliptic curve and vice versa do not satisfy, in general, this homomorphic property.

#### 2.2.2. An Application of Paillier Cryptosystem

This asymmetric cryptosystem was invented by P. Paillier in [11]. The public key is given by a pair (n,g), where *n* is the product of two large primes, n=pq, and *g* is a random element of $Z_{n^{2}}$. The corresponding private key is defined by the following parameters:

- λ=(p−1)·(q−1);
- $\mu ={\left(L(g^{\lambda }\;\left(\mathrm{mod}n^{2}\right)\right)}^{-1}\mathrm{mod}n$, where the function L(x) is defined as the integer quotient $\frac{x-1}{n}$.

Thus, the encryption function for an element *m* in $Z_{n}$ is defined by $E\left(m,r\right)=g^{m}\cdot r^{n}\mathrm{mod}n^{2}$ for some random element *r* in the rank {1,…,n−1}.

The original message *m* is recovered from the encrypted message *c* by using the private key in the decryption process as follows:

$$D\left(c\right)=m=L\left(c^{\lambda }\mathrm{mod}n^{2}\right)\cdot \mu \mathrm{mod}n$$

The security of this cryptosystem is based on the hardness of the problem on deciding whether an element x∈ 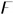 is an *n*-th residue module $n^{2}$ or not, i.e., decides if there exists $y\in Z_{n}$ such that $x=y^{n}\mathrm{mod}n^{2}$. The author in [11] shows that whenever the integer factorization problem is hard, then the underlying problem that ensures this cryptosystem is intractable.

In our setting, we are considering a set of sensor nodes $S_{1},\ldots ,S_{n}$ that constitute a device farm jointly with the corresponding bridge node *B* and that transmits the measured data $m_{1},\ldots ,m_{n}$ using the central server’s public key (n,g) of a Paillier’s cryptosystem. This will be denoted by $E(m_{i},r_{i})$, i=1,…,n. The bridge node *B* can provide information directly to the central server, acting as a virtual node, allowing this to obtain the mean and the variance of the set $\left\{m_{1},\ldots ,m_{n}\right\}$ by making simple computations on $E(m_{i},r_{i})$, i=1,…,n.

**Theorem** **1.** *Let $m_{i}$ be the data transmitted by the sensors $S_{i}$, i=1,…,n and let $\bar{m}$ and $\sigma^{2}$ be the mean and the variance of the preceding set of data, respectively. Then:*

(*a*) *$D\left(\prod_{i=1}^{n}E\left(m_{i},r_{i}\right)\right)=n\cdot \bar{m}$.*
(*b*)
$D\left(\prod_{i=1}^{n}E{\left(m_{i},r_{i}\right)}^{m_{i}}\right)=n\cdot \left(\sigma^{2}+{\bar{m}}^{2}\right)$

**Proof.**  (a) The proof follows by using the homomorphic properties of the cryptosystem: $\prod_{i=1}^{n}E\left(m_{i},r_{i}\right)=\prod_{i=1}^{n}g^{m_{i}}r_{i}^{n}=g^{\sum_{i=1}^{n}m_{i}}{\left(\prod_{i=1}^{n}r_{i}\right)}^{n}=E\left(\sum_{i=1}^{n}m_{i},\prod_{i=1}^{n}r_{i}\right)$. If we now apply the decryption function *D* and the definition of $\bar{m}$, the result is immediate.

(b) In the same way, we use the homomorphic properties of the cryptosystem:
  $\prod_{i=1}^{n}E{\left(m_{i},r_{i}\right)}^{m_{i}}=\prod_{i=1}^{n}{\left(g^{m_{i}}\right)}^{m_{i}}{\left(r_{i}^{n}\right)}^{m_{i}}=g^{\sum_{i=1}^{n}m_{i}^{2}}{\left(\prod_{i=1}^{n}r_{i}^{m_{i}}\right)}^{n}=E\left(\sum_{i=1}^{n}m_{i}^{2},\prod_{i=1}^{n}r_{i}^{m_{i}}\right)$. The result now follows by applying the decryption function and taking into account that $\sigma^{2}=\frac{\sum_{i=1}^{n}m_{i}^{2}}{n}-{\bar{m}}^{2}$.

□

From the preceding, it is clear, then, that if the sensor node in a device farm $S_{i}$, i=1,…,n sends the bridge node $E(m_{i},r_{i})$ and $E(m_{i}^{2},r_{i}^{\prime })$, then, by multiplying all these values, *B* can send to the central server the information necessary to compute $\bar{m}$ and $\sigma^{2}$. However, this forces $S_{i}$, i=1,…,n to encrypt two values, namely, $m_{i}$ and $m_{i}^{2}$. However, from (b) in the preceding Theorem, $S_{i}$ needs only to encrypt $m_{i}$ and then compute the corresponding power $E{\left(m_{i},r_{i}\right)}^{m_{i}}$, i=1,…,n.

In the above situation, the public information that is sent to *B* includes $a=E(m_{i},r_{i})$ and $b=E{\left(m_{i},r_{i}\right)}^{m_{i}}$, and if an external observer is able to solve the discrete logarithm problem $m_{i}=\log_{a}\left(b\right)$, then she would obtain the information $m_{i}$ without attacking the Paillier cryptosystem. An alternative to the proposal that depends uniquely on the underlying problem of the Paillier cryptosystem is that *B* shares with every $S_{i}$, i=1,…,n a secret value $s_{i}\in Z_{n^{2}}$ that is invertible and that $S_{i}$ sends the pair $(E\left(m_{i},r_{i}\right),s_{i}\cdot E{\left(m_{i},r_{i}\right)}^{m_{i}}$. Thus, *B* multiplies by $s_{i}^{-1}$ and obtains $E{\left(m_{i},r_{i}\right)}^{m_{i}}$, i=1,…,n.

## 3. Results

**Figure 3:**
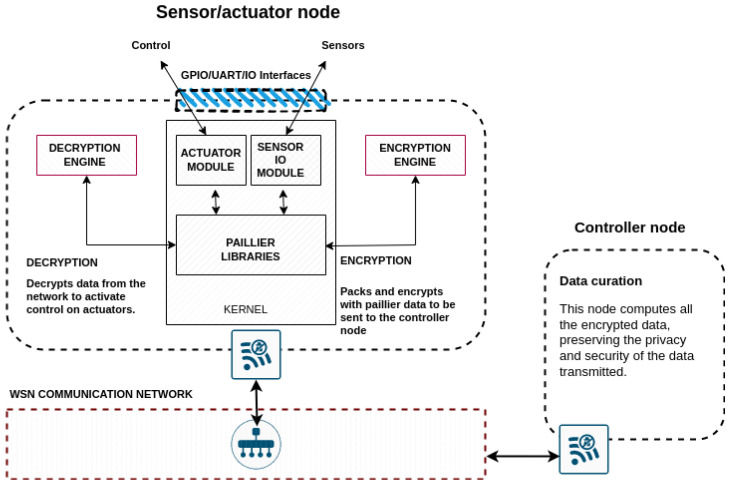
Computing infrastructure.

**Figure 4:**
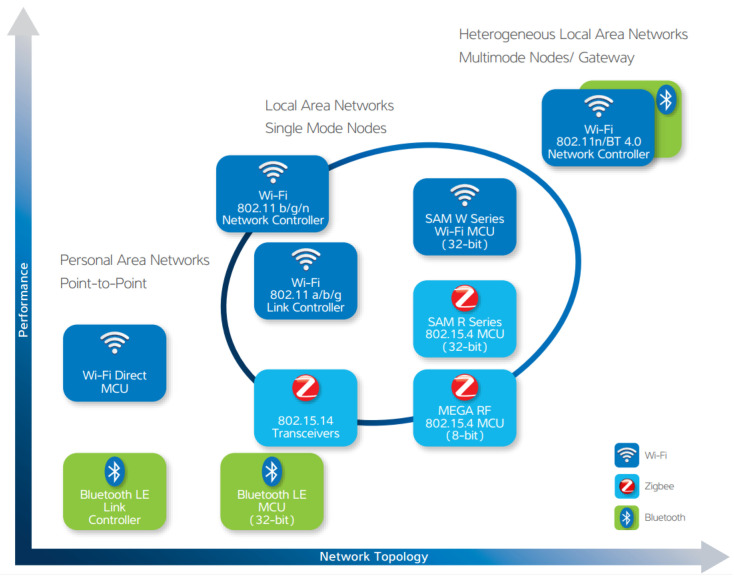
Networking capabilities.

Figure 3 shows the architecture of an IOT network node where Paillier is used as partially homomorphic encryption system. The node collects the information from the sensors, encrypts it, and sends it over the network to the bridge node that will perform the control and data curation tasks. Figure 3 describes the essential components of a node that is part of a wireless sensor network. In this sense, it must have input and output interfaces to connect the processor (or microcontroller) to the sensors. These (the sensors) are able to transmit information through these interfaces to the processor. All processors on the market, available for IoT applications, have analog-to-digital and digital-to-analog converters to process the information derived from the sensors. They must also have control connectivity (also through the input and output interfaces) to be able to act on the automated systems (if they are being used) based on the decisions made by this same node or by others in the network that assume control responsibilities based on the information sent by the sensors. If you look at Figure 3, you can see that another necessary feature in these nodes (as described in more detail in Figure 4) is connectivity with the other sensor nodes or with other (higher-level) nodes that allow for injecting the information from the sensors (once its quality has been checked) into a blockchain or deriving it to other business processes. Thus, a sensor node must have connectivity with sensors (if necessary, using DAC-ADC) with actuators and with a—preferably—wireless network environment. The most interesting capabilities of the node for IoT applications, likewise, are those that allow us to process the information received. Current ARM and similar processors have cores that are highly prepared to cope with AES ciphers and solve them efficiently (in terms of time and energy), a relevant example in this regard is the ARM Cortex-A57 processor Cryptography engine, which supports the ARMv8 Cryptography Extensions. The Cryptography Extensions add new instructions that the Advanced SIMD can use to accelerate the execution of AES, SHA1, and SHA2-256 algorithms. Therefore, the mathematical operations required to approach the homomorphic method we propose do not pose a difficulty for these architectures. Therefore, the information coming to these processors from the sensors can be encrypted, almost natively, if the processor has cryptographic extensions; if not, the repertoire of these processors allows them to perform the operations comfortably and to be able to send the information collected from the sensors, conveniently encrypted, to other nodes of the network or to the higher-level node. Likewise, if these nodes need to act on any system or automatism, they are prepared to decipher the decisions and act on the control mechanisms. In this sense, we call these blocks that allow cryptographic operations, in Figure 3, Decryption Engine or Encryption engine. All this supports the Paillier method and the libraries used.

Tests related to how the encryption and decryption performs and have been conducted once the protocol was implemented to asses its applicability. Such performance tests include measuring the time it takes for a node (we used three different architectures configuration in which we were able to confirm that the memory footprint of the method was minimal) to perform each operation.

The IoT application field is growing exponentially and with it the applications that will use the associated technology. Likewise, the technology that can be used in the IoT is advancing, driven by the need for emerging applications that have not yet found an answer with current devices. This implies that any experimental development must be tested on a well-known embedded platform that allows validation of key exchange protocols, etc.

Therefore, the aim is to avoid failures in developments and deployments unrelated to protocols and non-standard embedded technology (antennas, connectivity failures, premature battery consumption, failures in communication with sensors, errors in data processing, etc.). The authors in [18] intended to create a standard platform with which to test and design protocols, consisting of four layers:

- Communication;
- Processing;
- Power consumption;
- Sensorization.

All of them are ultimately the parameters that must be standardized when it is intended to expose the strength, as in the case of this work, of a communication protocol in which homomorphic cryptography is used. However, it is not only necessary to pay attention to the processor (or microncontroller) on which the protocols are to be tested, but also to the network architecture, which has evolved from a centralized model to a composite distributed model, as we have defined in Figure 1.

Therefore, to validate our protocol, and as stated in the literature, it is preferable to adopt the model defined in Figure 1. It is therefore necessary to use a testbed where the protocols developed in this work can not only be reproduced but also validated with sufficiently proven technology.

In the pool of widely accepted architectures used in the literature, and again relying on the paper in [18], we identify two large blocks of architectures that can be used in the IoT:

- Microcontrollers (UC Berkeyely Mica and similar "motes"), which are embedded platforms whose processing core is typically constituted by an atmega microcontroller, low-power radio modules and the ability to connect boards with sensors. The system they usually run is very restrictive and restricted, and in most cases, it is not useful to use cryptographic libraries (in this case, they should be implemented from scratch, and this incurs the risk of increasing vulnerabilities and running inefficient code).
- ARM, as in the case of iMote (Intel), or particular developments on ARM where there is a powerful, low-power-consumption, and well-proven architecture and where, in addition, the communication modules (antennas) are incorporated in the area dedicated to the processor [19].

The authors in [18] also clearly define the requirements of the processing and communication layer where the need for powerful digital signal processing units is emphasized, where ARM is undoubtedly an effective platform, and where it is also necessary to control the search of nodes and authorized neighbors with the ability to specify the efficient performance of concurrent tasks.

### 3.1. Trends

By powering the edge nodes that form the link between individual devices and the gateways that connect to the cloud, microcontrollers such as Atmel provide engineers with all the basic building blocks for Internet of Things (IoT) applications (from embedded processing and connectivity to sensors, security, and software) and tie it all together with a rich ecosystem of design tools and development partners. Such integrated platforms are a serious peer for ARM-based nodes. However, when it comes to embedded processing, Atmel avr or ARM-based microcontrollers deliver optimal combinations of performance and power efficiency.

#### 3.1.1. Security

As trust is a key element of the IoT, if applications are being developed in such an ecosystem, then it must be ensured that the data transferred back and forth have not been tampered with and that data are kept confidential. In this case, both ARM and Atmega incorporate Crypto-Authentication devices with protected hardware key storage. So, in terms of cryptography, the two options might be equivalent.

#### 3.1.2. Sensing

Sensors are essential parts of the IoT, giving connected devices the the ability to track and respond to environmental conditions. Sensing requirements have changed from simple monitoring to the simultaneous analysis and fusion of data from different sensors and sensor types. This includes accelerometers, gyroscopes, and magnetometers, as well as environmental sensors such as those detecting light level, color, temperature, pressure, humidity, etc. Both platforms are designed to operate concurrently with sensors and with the data provided from them.

#### 3.1.3. Communication

Efficient and effective wireless applications require standards-based technology, powerful transceivers that support a wide range of frequencies, as shown in Figure 4, and devices that strike an optimum balance between high-performance and low power use. Atmel, as well as ARM, wireless technologies cover multiple in-demand wireless arenas to enable IoT communication:

- 802.15.4 (Zigbee,® 6LoWPan);
- Bluetooth;
- Wi-Fi.

To help accelerate integration, both testbeds offer wireless solutions in SoCs (systems-on-chips) and modules.

### 3.2. Testbed

The testing architectures, as has been previously justified, are based on the ARM Cortex architecture. Testbed configurations are described below:

- *Testbed1*: Four 64-bit ARM operating at a clock rate of 1.5 Ghz, 4 GB RAM (Cortex A72);
- *Testbed2*: Four 64-bit ARM operating at a clock rate of 1.5 Ghz, 128 MB RAM (Cortex A72);
- *Testbed3*: Four 64-bit ARM operating at a clock rate of 1.2 Ghz, 1 GB RAM (Cortex A53).

The addition and multiplication operations in the Paillier scheme for 64-bit in the testbed architectures showed to be feasible, as sequential homomorphic operations have been conducted on the encrypted data that are modeled as the sensor input. Such tests were executed on *Testbed3* architecture, as it configures the slower baseline of the method.

Once the operational tasks are proven to be feasible, the complete encryption and decryption operations for the homomorphic Paillier method have been timed, and the results can be observed in Table 1, where the encryption used a key of 2048 bits in length.

The use of homomorphic cryptography in the IoT is possible; not only is it possible, but it is being applied [20,21,22,23,24]. The results in Table 1 show times in the same orders of magnitude (but lower) than those shown in other recently published work using homomorphic cryptography. In [20], the authors use similar processor architectures (processor clocks in a range varying from 1.4 to 2.5 GHz and with a memory configuration from 4 GB to 8 GB). With such configurations, they achieve with a Google Pixel 3 smartphone (we can understand this device as fitting the conditions of an IoT device as depicted in Figure 3 in terms of comparing encryption times) an encryption time of 0.609 s. In [22], the authors included a RaspberryPi equipped with a ARM Cortex A53 at 1.4 GHz, showing a performance of 4.13 milliseconds per byte, in this case, a node gathering a 64-bit-long integer or double per second is capable of dealing with the packing and encrypting of the data.

As a reference, the RSA homomorphic encryption on *Testbed 1*, which is a less versatile method, took approximately 1 millisecond to encrypt and 10 milliseconds to decrypt. The results obtained with the Pailler encryption and decryption methods are shown to last longer, but given their versatility, they are suitable for an IoT node. The private data aggregation process’s required costs are highly dependent on the key size used in the homomorphic encryption. A reduced key would accelerate the process but would also decrease the security level. We have tested our method with 2048-bit-length keys, but regarding the needs of the deployment of every particular IOT WSN setup, a trade-off between computation requirements and security needs to be configured.

## 4. Discussion

We have derived two simple formulae that allow us to compute statistical measures from encrypted information using a Paillier cryptosystem. This allows us to analyze in real time the information received from the sensor nodes. We have considered two different possible architectures.

On one hand, we have the architecture of [6], where nodes are distributed in device farms, each one containing a detached node, called the bridge node, in charge of packaging the information previously to be sent to the nodes that verify the information and add it to a private blockchain. By using this technique, bridge nodes can compute the necessary information to analyze the transmitted data from the sensor nodes without decryption. The usual mode of communication in this group is to use a session group key shared by the nodes in the device farm (the sensor nodes and the corresponding bridge node) in order to achieve confidentiality and avoid possible misinformation and/or information leakage. If the bridge node is in charge of computing the information necessary to obtain the statistical data, then it should decrypt every sensor node message and then compute the corresponding statistical measure. Once this is completed, all the messages are packed jointly with the statistical information and sent to the sensor node or server in charge of analyzing the data and adding it to the blockchain. Our proposal allows the statistical information to be computed from the same moment that the bridge node starts receiving messages from the different nodes, without waiting to receive the whole set of messages and decrypt them, since this node can multiply every received message by the product of all the previously received messages, which speeds up the process significantly and reduces the workload of the bridge nodes, using much less storage and computing resources. Secondly, the messages that correspond to the statistical measures allow the nodes in charge of analyzing the information or the central server to obtain information from the whole device farm without decrypting every message, just those corresponding to the statistical information, which allows us to have an idea of the device farm’s functioning just with a simple decryption. This, in turn, allows us to compare a particular device farm with the rest. This gives the possibility of detecting the incorrect functioning of a bridge node or a whole device farm by comparing the corresponding statistical information with the statistical information of the rest of the device farms. Moreover, the bridge nodes do not have access to the transmitted data since this is encrypted in such a way that only the nodes or server in charge of checking and analyzing the data can access them.

On the other hand, we can consider an architecture given by groups of nodes that communicate directly with a a central server. We will include here a node that will act as a virtual sensor node in every group. Here, we would consider a situation where every sensor node sends the encrypted data twice: one first message directly to the central server and a second one to the virtual node. The information is encrypted by using the key of the central server, so only this can access the data as previously described, i.e., the virtual node does not have any information on the collected data in its group. The virtual node computes in real time the encrypted statistical measures of its group by using the derived formulae, and these are sent to the central server as data that the virtual node has collected as well. All the preceding advantages concerning storage, use of computing resources, as well as speeding up of the statistical analysis also apply in this case.

## Figures and Tables

**Table 1:** Results (milliseconds).

	Testbed 1	Testbed 2	Testbed 3
**Encryption**	101.45	115.81	556.02
**Decryption**	113.62	127.79	601.35

## Data Availability

Not applicable.
